# A Patient with Graves' Disease Scheduled for Thyroidectomy with High Risk for Thyroid Storm Caused by Severe Medication Nonadherence: Anaesthetic and Surgical Considerations

**DOI:** 10.1155/2019/4781902

**Published:** 2019-07-22

**Authors:** Adrian Reber, Laura Valenti, Stephan Müller

**Affiliations:** ^1^Department of Anesthesiology and Intensive Care Medicine, Hospital of Zollikerberg, 8125 Zollikerberg, Zurich, Switzerland; ^2^Department of Surgery, Hospital of Zollikerberg, 8125 Zollikerberg, Zurich, Switzerland

## Abstract

In patients with failed hormone regulation who are scheduled for indispensable total thyroidectomy, the risk of thyroid storm with severe end-organ complications has to be anticipated. This case report presents the successful surgical and anaesthesiological management of a patient with Graves' disease, without any signs of perioperative thyroid storm. Possible recommendations for treatment are presented.

## 1. Introduction

Graves' disease is one of the most common causes of hyperthyroidism. Thyroid-stimulating hormone (TSH) receptor antibodies in the blood stimulate the thyroid gland, resulting in diffuse thyroid enlargement. Patients exhibit suppression of pituitary TSH and increased levels of thyroxine (T4) and/or triiodothyronine (T3) [1]. The primary goals before surgery are creating a euthyroid state, assessing the degree of end-organ complications, and determining the extent of airway involvement [2].

For elective surgery with preceding successful treatment of hyperthyroidism, risk of hormone deterioration in the form of a thyroid storm is unlikely. A thyroid storm is characterized by a rapid increase in circulating T3 and T4, tachycardia, hypertension, and hyperthermia [3]. Inorganic iodide and antithyroid drugs are recommended in patients with thyroid storm caused by thyrotoxic diseases associated with hyperthyroidism [4]. We report a case of successful management of a patient with Graves' disease and failed hormone regulation due to severe medication nonadherence who was scheduled for indispensable total thyroidectomy.

## 2. Case Presentation

A 23-year-old woman (height, 165 cm; weight, 65 kg) with Eurasian background presented with dyspnoea on exertion, notable tremor, bulging eyes, enlarged thyroid gland, hypertension (blood pressure, 150/90 mmHg), and tachycardia (128 beats/min) to a general practitioner. The patient was diagnosed with bronchial asthma at the age of twelve and is medicated with salbutamol and budesonide/formoterol. Biochemical investigations revealed a derangement of thyroid function in terms of Graves' disease (Table 1). Thyrotropin receptor antibodies showed high titres of 25.29 U/L. The patient was started on carbimazole (up to 80 mg/day) and propranolol (40 mg, 4 times a day) by a practitioner. Although beta blockers are generally contraindicated in patients with bronchial asthma, they can be used cautiously in patients in whom heart rate control is essential under careful monitoring of pulmonary status [5]. After two months on carbimazole, the patient was admitted to the university hospital for evaluation by an endocrinologist. The patient showed dyspnoea on exertion as well as hyperthyroidism-related mental instability, and blood hormone levels remained high. Therefore, thyrostatic therapy was changed to propylthiouracil (50 mg, 3 times a day). Radioactive iodine therapy was not considered because of the risk of worsening of Graves' orbitopathy [6]. A low-iodine diet was prescribed. However, after 10 days propylthiouracil treatment was stopped because the patient suffered from adverse effects of nausea and vomiting. Carbimazole was restarted despite persisting sickness. Blood hormone levels improved and were half of the initial levels (Table 1). However, the results remained high. The volume of the enlarged thyroid gland was estimated as 35 mL by using ultrasound (threshold value, 18 mL). Thyroidectomy was urgent because of severe hyperthyroidism-related severe mental instability, noncompliance of the patient, and threat of therapy withdrawal by the patient. Due to the urgency of thyroidectomy and treatment nonadherence in the present affective disorder, the patient could not be prepared with intravenous glucocorticoids prior to surgery. The patient was scheduled for surgical and anaesthesiological evaluation in another hospital. She suffered from endocrine orbitopathy and complained of diplopia and blurry vision. Severe hyperthyroidism-related mental instability and anxiety and tearfulness were noted, with a high level of suicide risk.

Premedication consisted of oral administration of midazolam 7.5 mg. The patient was less agitated than the previous day, and her pulse rate normalized to 80 beats/min. Her blood pressure was 120/80 mmHg. General anaesthesia was induced through intravenous administration of midazolam 4 mg, sufentanil 20 *μ*g, and propofol with a high target plasma concentration of 8 *μ*g mL^–1^ over 3 min by using the Arcomed infusion device (Arcomed Medical Systems, Regensdorf, Switzerland). The depth of anesthesia was monitored through bispectral index, BISx, using the Aspect Medical Systems' XP platform technology (Philips Medical Systems, Eindhoven, The Netherlands). When the patient lost consciousness with this initial target plasma concentration, a radial arterial catheter was placed to enable continuous blood pressure measurement. Muscular paralysis was induced after intravenous administration of rocuronium (25 mg). Prior to endotracheal intubation, hydrocortisone (100 mg) and esmolol (30 mg) were administered intravenously. Endotracheal intubation was effortlessly performed using a cuffed tube (inner diameter, 7.0 mm) in the first attempt through video laryngoscopy with a C-MAC blade (Karl Storz Company, Tuttlingen, Germany). External manipulation of the neck was avoided. The lungs were mechanically ventilated at a rate of 12–14 bpm with 60% oxygen in nitrogen with a Flow-I ventilator (Maquet, Rastatt, Germany). Minute volume was adjusted to maintain end-tidal carbon dioxide concentration at 4.5 and 5.0 kPa. Target plasma concentrations of propofol were titrated to maintain low BIS target values (between 6 and 10) throughout the intraoperative period. In addition, midazolam was administered repetitively as an antiepileptic drug. During the thyroidectomy, squeezing and mobilization of the thyroid gland was avoided, and accurate bipolar coagulation was performed.

Recurrent laryngeal nerve was monitored through endotracheal tube electrodes by using the Avalanche Novimed system (Dr Langer Medical, Waldkirch, Germany) throughout the surgical procedure, which lasted 2 h and 15 min. The patient remained hemodynamically stable without episodes of tachycardia or cardiac arrhythmias. However, BIS values highly fluctuated with sharp and sudden increases up to 70 within seconds, despite uncommonly high amounts of anaesthetics (propofol, 3274 mg; sufentanil, 250 *μ*g; and midazolam, 78 mg in total). Therefore, tracheal extubation was delayed, and the patient was transferred to an intensive care unit; tracheal extubation was performed after 3 h. Subsequently, the patient showed hyperthyroidism-related mental instability and noncooperation. The next day, a computed tomography scan of the head was performed because the patient experienced formication and sensory disturbances, including weakness and convulsions of the right arm as well as dyspnoea. Imaging did not reveal any pathology, and the symptoms were interpreted as a part of a hyperventilation episode. Carbimazole and propranolol medications were stopped. The patient was discharged on the third postoperative day. The results of biochemical investigations are presented in Table 1.

Histology revealed benign tissue samples. Seven days postoperatively, hormone substitution was initiated with levothyroxine. Possible adjustments of medication were defined, with the objective of achieving a clinically euthyroid state with TSH levels between 0.5 and 2.5 mU/L.

## 3. Discussion

Patients with Graves' disease presenting for any type of surgery should ideally be biochemically and clinically euthyroid [6]. In our patient, antithyroid drugs and beta blockers did not relieve the symptoms of thyrotoxicosis, and thyroid hormone levels remained high. Despite endocrine imbalance, no manifestation of uncontrolled and severe derailment occurred. Some cornerstones of patient management are highlighted and discussed here.

Critical circumstances such as the adverse side effects of antithyroid drugs and noncompliance with a medication can lead to uncontrolled thyrotoxicosis. The risk of life-threatening complications should be compared with the risk of anaesthesia and surgery. A patient may prefer surgery in order to avoid the adverse effects of antithyroid medication, to avoid radioiodine, and to obtain rapid correction of the endocrine system. In general, medication nonadherence is a missed opportunity for therapeutic benefit and for a patient's protection. In our patient with severe malcompliance, surgery could not be postponed because of intolerance to medication and severe hyperthyroidism-related mental instability with a high level of suicidality. Occurrence of intraoperative [7] and postoperative [8] thyroid storm has been reported prior to the induction of anaesthesia [9]. Inorganic iodide should be administered simultaneously with antithyroid drugs to patients with thyroid storm caused by thyrotoxic diseases associated with hyperthyroidism (strength of recommendation: high; quality of evidence: moderate) [4]. Thus, in the present case, to avoid the aggravation of the patient's preoperative condition, particular care was required. Gaining the patient's confidence, adequate premedication, and a quiet environment is crucial prior to the induction of anaesthesia. Because thyroid size and dyspnoea and globus sensation are positively correlated [10], a ramped positioning of the head is required in patients with a large thyroid gland.

Corticosteroids should be administered as prophylaxis for relative adrenal insufficiency caused by the hypermetabolic state in the case of thyroid storm (strength of recommendation: high; quality of evidence: moderate) [4]. Thyroid storm predisposes a patient to develop supraventricular tachycardia, which can lead to severe cardiopulmonary dysfunction [11]. Thus, invasive blood pressure measurement is highly recommended. Extreme hemodynamic changes after injecting glycopyrrolate have been reported in a patient with hyperthyroidism [12].

As excessive amounts of thyronines shorten the biological half-life of the beta adrenergic blocking agents, long-acting and complementary short-acting beta blockers are highly recommended in patients with trophoblastic hyperthyroidism [9]. For immediate hemodynamic regulatory interventions, esmolol infusions (up to 200 *μ*/kg/min) are advocated [9]. However, propranolol alone does not prevent thyroid storm [13]. In the rapid preoperative preparation of a patient with severe thyrotoxicosis, the safety and effectiveness of beta blocking agents can be increased using corticosteroids up to the day of surgery [14]. In our patient, hydrocortisone was administered after the induction of anaesthesia.

Thyroid storm is nearly ruled out in well-medicated patients with Graves' disease. However, severe thyrotoxicosis, which can be triggered by anaesthesia and/or surgery/manipulation of the thyroid gland, exhibits a high mortality rate of approximately 10% [15, 16]. Therefore, external manipulation of the neck during intubation should be avoided. Endotracheal intubation should be effortlessly performed. During surgery, tissue compression and luxation of the vital thyroid gland should be avoided. Surgical excision requires an adequate transverse neck incision and accurate coagulation.

Low BIS values were achieved to avoid any stress response with a concomitant risk of thyroid storm [1]. However, in this case report, equilibrating our patient with anaesthesia was difficult because BIS values showed a very wide range with sharp and sudden increases. No changes in body temperature and haemodynamics occurred. In patients with intraoperative thyroid storm, an abrupt rise in body temperature, mimicking malignant hyperthermia, has been reported [8, 17]. Tonic-clonic seizures associated with thyroid storm have been reported [18]. Thus, for raising the potential seizure threshold, midazolam was repetitively administered as an antiepileptic drug. It is also reasonable to perform endotracheal extubation in a quiet environment.

In conclusion, possible recommendations for minimizing the risk of thyroid storm in patients with Graves' disease and failed hormone regulation who are scheduled for indispensable total thyroidectomy include the following:

- Minimizing any risk for stress response, including anaesthesiological and surgical precautions
- Use of long-lasting and short-acting beta adrenergic blocking agents in combination with corticosteroids
- Close communication between the surgeon and the anaesthetist
- Adequate patient monitoring, including invasive blood pressure measurement and BIS monitoring
- Considerably enhanced depth of anaesthesia and application of antiepileptic drugs
- Postoperative monitoring of the patient in an intensive care unit
- Awareness of the possibility of life-threatening pre-, intra-, and even postoperative thyroid storm

## Figures and Tables

**Table 1:** Results of biochemical investigations and clinical course of the patient. TRAK = thyrotropin receptor antibodies: U/L; FT3.

	–63 days	–23 days	–13 days	–5 days	Surgery	+1 day	+2 days	+7 days
	Start medication	Switch medication	Switch medication	Anaesthesiol. consultation	Prior to surgery	Follow-up	Follow-up	Follow-up
FT3	44.1		22.0	24.3	19.6			4.2
(3.0–6.5)								
FT4	99.7		41.9	44.5	47.1			18.9
(10.0–20.5)								
TSH	<0.01		<0.01	<0.008	<0.008			<0.002
(0.200–4.000)								
TRAK	25.29							
Heart rate	80		87	114	80	93	76	90
Blood pressure	110/80		128/75	150/85	120/75	130/55	140/94	130/85
